# Sterility in the Woman and Its Treatment

**Published:** 1893-09

**Authors:** 


STERILITY IN THE WOMAN AND ITS TREATMENT, BY DR. DE SINETY. Translated by E. P. Hurd, M. D. George S. Davis, 1893.

One of the Physicians' Leisure Library, which treats of a subject of interest to the Gynæcologist, and from the reading of which he may get useful hints for the treatment of some few of the ills to which woman is heir.

